# Cantilever Type Acceleration Sensors Made by Roll-to-Roll Slot-Die Coating

**DOI:** 10.3390/s20133748

**Published:** 2020-07-04

**Authors:** Sang Hoon Lee, Sangyoon Lee

**Affiliations:** 1 Department of Mechanical Design and Production Engineering, Konkuk University, Seoul 05029, Korea; hoon1911@konkuk.ac.kr; 2 Department of Mechanical Engineering, Konkuk University, Seoul 05029, Korea

**Keywords:** acceleration sensor, cantilever, roll-to-roll slot-die coating, air gap, sacrificial layer

## Abstract

This paper presents the fabrication by means of roll-to-roll slot-die coating and characterization of air gap-based cantilever type capacitive acceleration sensors. As the mass of the sensor moves in the opposite direction of the acceleration, a capacitance change occurs. The sensor is designed to have a six layers structure with an air gap. Fabrication of the air gap and cantilever was enabled by coating and removing water-soluble PVA. The bottom electrode, the dielectric layer, and the sacrificial layer were formed using the roll-to-roll slot-die coating technique. The spacer, the top electrode, and the structural layer were formed by spin coating. Several kinds of experiments were conducted for characterization of the fabricated sensor samples. Experimental results show that accelerations of up to 3.6 g can be sensed with an average sensitivity of 0.00856 %/g.

## 1. Introduction

Acceleration sensors are usually fabricated using microelectromechanical systems (MEMS) technology [1,2,3,4,5]. They have been applied to the fields of automobile [1,5], healthcare [2], mobile devices [3], and electronic devices [4]. MEMS acceleration sensors are divided into capacitive [6,7], piezoelectric [8,9], piezoresistive [10,11], Hall effect [12,13], magnetoresistive [14,15] and heat transfer [16,17] types according to the sensing method used. The most common type of MEMS acceleration sensors are capacitive because of their simple structure, high productivity, linear stability, durability, and insensitivity to temperature [18,19].

Capacitive acceleration sensors can have a cantilever [20,21], sandwich [22,23] or comb [24,25] structure and the structures are made to contain an air gap. Air gaps are commonly formed by etching in MEMS [26,27]. When an acceleration occurs in a capacitive acceleration sensor, the mass placed on or beside the air gap moves in the opposite direction of the acceleration by inertia. As a result, the capacitance changes by which the acceleration is measured.

**Figure 1:**
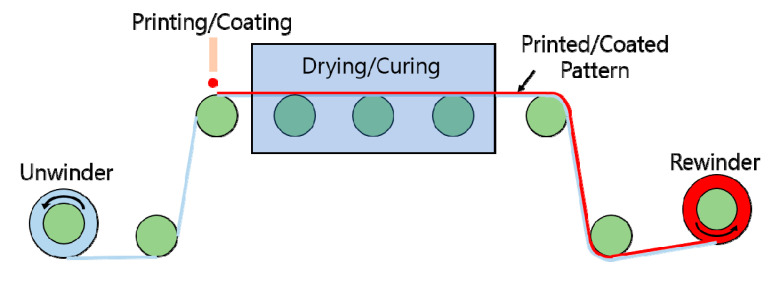
Schematic of a roll-to-roll gravure printer.

Printed electronics [28,29,30,31,32,33,34] is considered a promising alternative technology to MEMS because of its advantages in productivity, cost, and suitability for fabricating flexible electronic devices. Numerous printing methods such as gravure [35,36,37,38], offset [39,40,41] and flexograpy [42,43,44] are used. In addition, coating methods such as slot-die coating [45,46,47,48] and spin coating are also employed. Among the various printed electronics processes, the roll-to-roll printing process [49,50,51] has the highest productivity. While a substrate moves continuously from the unwinder to the rewinder in the roll-to-roll process, it experiences printing/coating and drying (Figure 1).

However, devices with air gaps are difficult to fabricate in printed electronics, where no process corresponding to etching in MEMS is available. MEMS processes often involve lithography-based processes and require more than five steps, including deposition, exposure, developing, and wet/dry etching, but the printed electronics process consists of only two: printing (or coating) and drying, without etching.

Accordingly, very few works on air gap-based sensors are found in the printed electronics field. Air gaps are often made in printed electronics by using a wet etching-like process: printing/coating followed by removal of the sacrificial layer, but most materials used for MEMS wet etching are not suitable because the fabrication process in printed electronics is often conducted under low temperature (<200 °C). Therefore, finding suitable materials for printed electronics processes is a significant issue. Some examples are motion sensors [52], touch sensors [53] and switching devices. [54] An air gap-based bridge type touch sensor was fabricated and reported by the authors [55], but studies on the fabrication of air gap acceleration sensors have not been reported and fabrication of air gap-based electronic devices using the productive roll-to-roll process has not been conducted either. The roll-to-roll printing process is difficult to optimize compared to the sheet-to-sheet approach using ordinary printing processes including screen printing, inkjet printing, and spin coating. However, the roll-to-roll process is considered as a commercially attractive solution because of its high productivity in terms of cost, scale, and mass production. Therefore, studies for fabricating sensors using the roll-to-roll process need to be continued.

**Figure 2:**
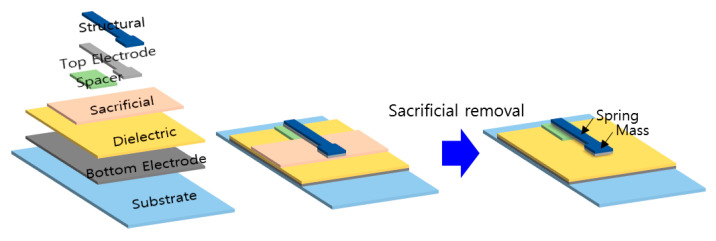
Schematic of the cantilever type acceleration sensor.

This paper presents fabrication of air gap-based cantilever type acceleration sensors using the productive roll-to-roll slot-die coating method. Figure 2 shows a schematic of the sensor, composed of several layers. The bottom electrode, the dielectric layer, and the sacrificial layer were formed by the roll-to-roll slot-die coating method. The spacer, the top electrode, and the structural layer were made by spin coating.

As for selecting proper inks for the spin coated layers, extensibility to roll-to-roll gravure printing for further studies was also considered. Inks with low viscosity and high surface energy were chosen such that they can be used under low temperature (<150 °C), which is necessary for roll-to-roll gravure printing. Water-soluble PVA [56] was used as a material for the sacrificial layer. Since PVA can be removed with water only, the removal process has little effect on the other layers. Coating and removing PVA yields the structures of the air gap and cantilever.

The air gap-based cantilever type acceleration sensors fabricated in this study are capacitive. When the sensor is under an acceleration, the spring and the mass, i.e., the top electrode and the structural layer in Figure 2, move in the opposite direction of the acceleration. Then the capacitance between the top and the bottom electrodes changes, by which the sensor detects the acceleration.

## 2. Materials and Methods

**Figure 3:**
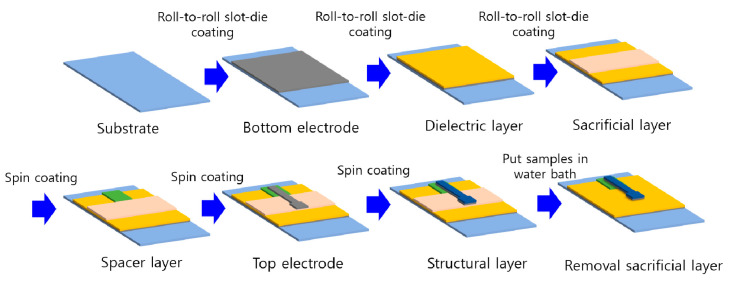
Fabrication sequence of the cantilever type acceleration sensor.

Air gap-based cantilever type capacitive acceleration sensor samples were fabricated using two coating methods: roll-to-roll slot-die coating and spin coating. The bottom electrode, the dielectric layer, and the sacrificial layer were formed using the productive roll-to-roll slot-die coating (Naraenanotech, Korea). The spacer layer, the top electrode, and the structural layer were formed by spin coating (Midas system, Korea). The fabrication sequence is shown in Figure 3. The roll-to-roll slot-die coater consists of an unwinder, a rewinder, a slot-die coater, an infrared oven, and a syringe pump. As a substrate moves continuously from the unwinder to the rewinder, ink that is injected into the slot-die by the syringe pump is coated on the substrate. A coated sample batch moves to the infrared oven, which is 5 m long, and then it is dried.

The bottom electrode was formed by roll-to-roll slot-die coating of silver ink (TEC-CO−021, InkTec, Korea) on a flexible polyethylene terephthalate (PET) substrate (SH34, SKC, Korea). Then ink was dried for 10 min at 100 °C in an infrared oven that is installed in the slot-die coater. The dielectric layer was formed above the bottom electrode to avoid shorting between the bottom and the top electrodes. Poly(methyl methacrylate) (PMMA) solution was used as a material for the dielectric layer. PMMA solution was prepared by dissolving 10 wt% of PMMA powder (120,000 g/mol; Sigma Aldrich, St. Loise, Missouri, USA) in acetone with a magnetic stirrer in the room temperature for 3 h. PMMA solution was roll-to-roll slot-die coated and dried in the infrared oven for 10 min at 70 °C.

**Figure 4:**
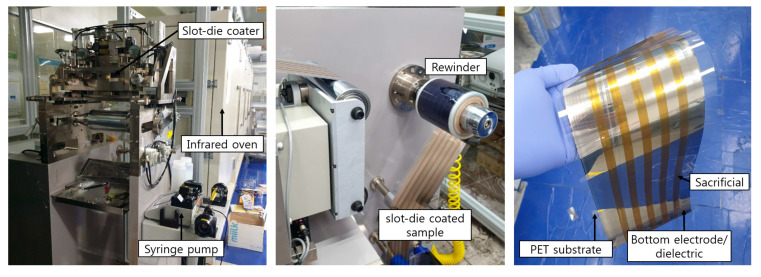
Photograph of a roll-to-roll slot-die coater (**left)**, rewinding of the coated and dried sample (**middle**), and samples of the bottom electrode, the dielectric, and the sacrificial layer (**right**).

PVA was used as a material for the sacrificial layer. PVA solution was obtained by dissolving 15 wt% of PVA powder (63,500 g/mol; Comscience, Korea) in water with a magnetic stirrer in the room temperature for 24 h. The solution was roll-to-roll slot-die coated and dried in the infrared oven for 10 min at 70 °C. The width and the thickness of the sacrificial layer were designed to be 7 mm and 100 μm, respectively. During the roll-to-roll coating of the three layers, the line speed was set at 0.5 m/min. The roll-to-roll slot-die coater and the roll-to-roll slot-die coated sample batch of the bottom electrode, the dielectric layer, and the sacrificial layer are shown in Figure 4. Sensor samples were taken from the batch for spin coating of the other upper layers, i.e., the spacer, the top electrode, and the structural layer.

Another layer called the spacer is added in order to avoid stiction that is a problem occurring when the top electrode collapses and attaches to the substrate. The problem can often happen during the removal of the sacrificial layer because the process is similar to the wet etching process in MEMS. The spacer layer was formed beside the sacrificial layer by spin coating of barium sulfate (BaSO_4_) ink (SOC2808, Toyobo, Japan) at 7000 rpm for 1 min and drying on a hot plate for 30 min at 150 °C.

Then the top electrode was formed above the sacrificial and the spacer layer by spin coating of stretchable silver ink (SSP2801, Toyobo) at 7000 rpm for 1 min and drying on the hot plate for 30 min at 150 °C. The top electrode is designed to have a structure of mass and spring. (see Figure 3) The stretchable silver ink has advantages in terms of less cracking and high restoration and elastic forces, compared to common silver ink. Such characteristics help restoration to the initial state of the top electrode after the sensor experiences acceleration.

Next, epoxy resin (F-301, Alteco, Japan) with the same shape and size of the top electrode was spin coated at 7000 rpm for 1 min and dried on the hot plate for 10 min at 70 °C to form the structural layer. The structural layer prevents the stiction problem as well as collapse of the top electrode after repeated occurrences of acceleration.

As a result of spin coating, the entire surface of film is usually covered with ink. In order to make the spin coated layers (the spacer, the top electrode, and the structural layer) have a specific shape, polyimide (PI) masks were employed. Specific patterns for the spin coated layers were designed with AutoCAD and sticky PI films were patterned with CraftROBO (Graphtec, Japan) to make the PI masks. Then the masks were attached to the samples. After inks were spin coated and dried, PI masks were removed.

**Figure 5:**
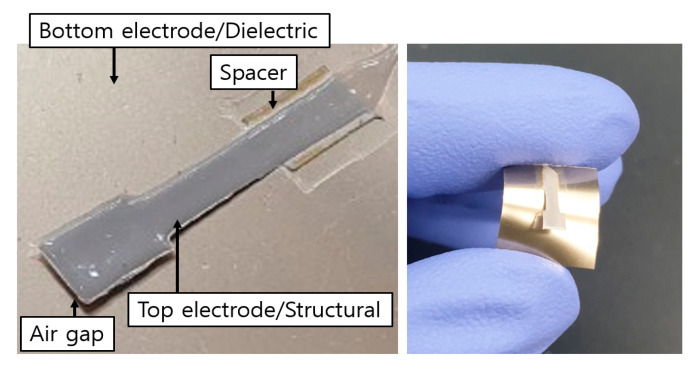
Photograph of a roll-to-roll slot-die coated cantilever type sensor sample.

Finally, sensor samples were put into a water bath for 1 h such that air gap can be formed by removing the PVA sacrificial layer. One of the completed sensor samples is shown in Figure 5. In addition, materials and fabrication processes for each layer are listed in Table 1.

The width and the thickness of the air gap were measured using a field emission scanning electron microscopy (FE-SEM; SU8010, Hitachi, Japan) and electrical characteristics of the sensor samples were examined using a homemade acceleration test equipment. The acceleration test equipment is composed of two Arduino modules (SZH-EK002, Ntrex, Korea), two Bluetooth modules (HC-05, Ntrex, Korea), a capacitance to digital conversion module (AD7745ARUZ, Analog Devices, Norwood, Massachusetts, USA), a BLDC motor (BL42S-24026N, D&J With, Korea), and a power supply (Protek 3032B, GSI, Korea).

## 3. Results and Discussion

FE-SEM images of the sensor samples were taken, and the width and the thickness of air gap were measured. Three sets of experiments were conducted to examine electrical characteristics of the samples: relationship between acceleration and capacitance, hysteresis characteristic, and repeatability. Three sensor samples in one batch were measured for each set of experiments for the characterization and the average values are represented as the rate of capacitance change (ΔC/C_0_ (%)).

### 3.1. Air Gap and Dimension of the Sensor Samples

**Figure 6:**
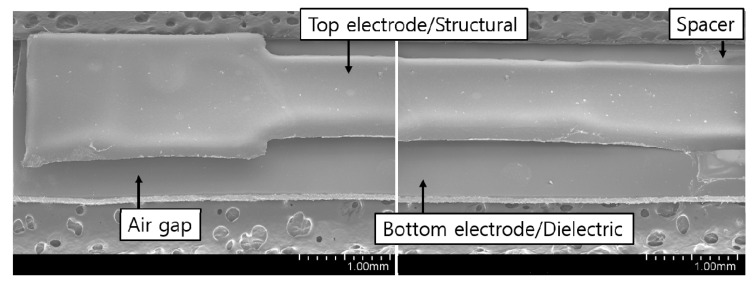
FE-SEM image of an air gap-based cantilever type capacitive acceleration sensor sample.

Figure 6 shows one of the FE-SEM images of the sensor samples. The mean of the width and the thickness of air gap are 7 mm and 108.7 μm, respectively, which is nearly identical to the design. The FE-SEM images also show that the samples do not have a problem of collapse and stiction of the top electrode as well as PVA residue.

### 3.2. Electrical Characteristics of the Sensor Samples

The cantilever type acceleration sensor is a kind of capacitive sensors [57,58], and it follows the capacitor characteristic in the following equation [59,60]:

(1) $$C=\frac{\varepsilon_{0}\cdot \varepsilon_{r}\cdot A}{d}$$

where *C* is the capacitance, *ε*_0_ is the electric permittivity of vacuum, *ε_r_* is the relative permittivity of dielectric, *A* is the area, and *d* is the thickness of the dielectric. The parameter *ε*_0_ is known as a constant 8.85 × 10^−12^ F/m. In this work, the air gap is equivalent to the dielectric of the acceleration sensor samples, and so *ε_r_* is 1.0006 and *d* is 108.7 μm.

**Figure 7:**
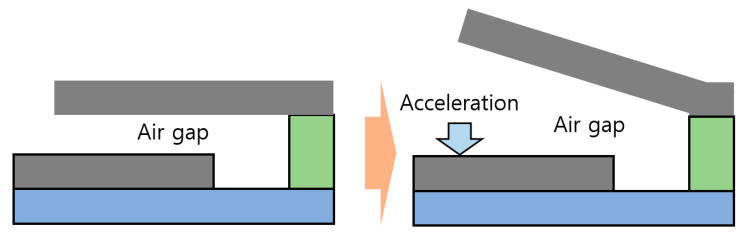
Thickness change of air gap by occurrence of acceleration.

When the thickness of air gap decreases, capacitance of the sensor increases. If the cantilever type sensor experiences acceleration in the specified direction in Figure 7, upper layers including the mass move in the opposite direction. As a result, the thickness of air gap increases, and capacitance decreases accordingly.

**Figure 8:**
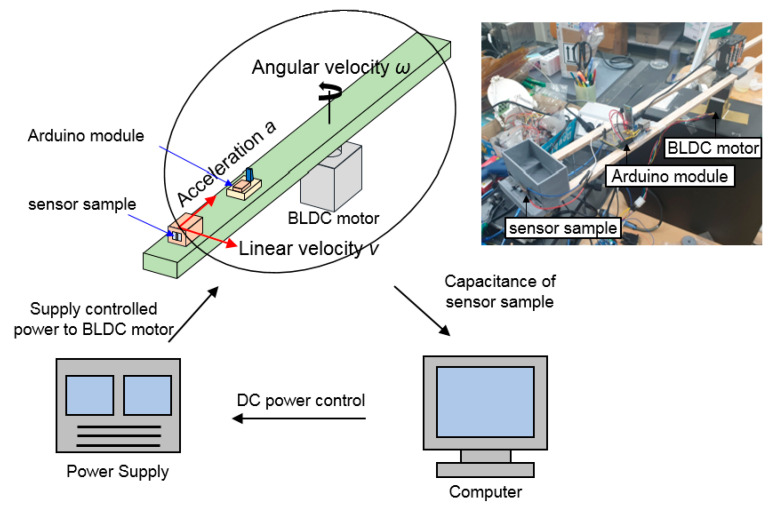
Layout of an experiment setup for measuring the capacitance of the sensor sample using the homemade test equipment.

Figure 8 shows a layout of an experiment using the homemade test equipment. An acceleration sensor sample is placed at the end of the beam. The bottom and the top electrodes are connected to the capacitance to digital conversion module that is installed in one Arduino module. Electric power from the power supply to the BLDC motor is controlled by a program in the computer. According to the signal to the motor, the beam rotates, and the sensor is under the centripetal acceleration. The centripetal acceleration can be determined by setting the angular velocity of the beam. The relationship between the acceleration and the velocity is based on the following equations [61,62]:

(2) v=r⋅ω,

(3) $$F=m\cdot \frac{v^{2}}{r},$$

(4) $$a=\frac{F}{m}=\frac{v^{2}}{r}=r\cdot \omega^{2},$$

where *v* is the linear velocity, *r* is the radius, *ω* is the angular velocity, *F* is the force, and *a* is the centripetal acceleration. The radius *r* is the length of the beam on the homemade test equipment, and it is 50 cm.

While the beam rotates, capacitance of the sample is acquired by the computer through the capacitance to digital conversion module. Accelerations of up to 3.6 g can be obtained using the equipment.

**Figure 9:**
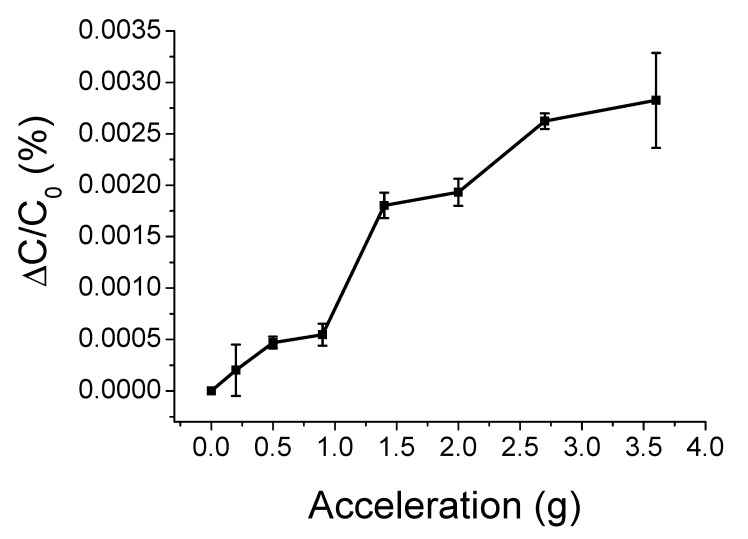
Relationship between the acceleration and the capacitance change of the cantilever type acceleration sensor samples.

First, the relationship between the acceleration and the capacitance change (ΔC/C_0_ (%)) was examined as acceleration increases from 0 to 3.6 g. As shown in Figure 9, the average capacitance change increases according to the increase of acceleration, and it reaches 0.00283% when the acceleration is 3.6 g. The average capacitance of the sensor samples at the initial state is 4.36 pF. The standard deviations of the average capacitance change are quite small for the entire range of acceleration except the end value of 3.6 g where the standard deviation is 0.00046. In addition, the sensitivity for the range of acceleration is 0.00856 %/g.

**Figure 10:**
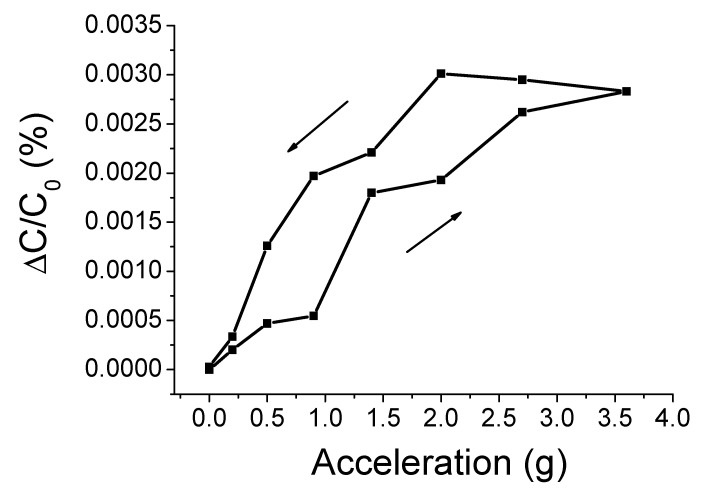
Hysteresis of capacitance change of the cantilever type acceleration sensor samples for the range of acceleration from 0 to 3.6 g.

Next, hysteresis of capacitance change of the sensor samples was examined. Capacitance was measured upward and downward for the same range of acceleration between 0 and 3.6 g. The result is shown in Figure 10. The hysteresis and maximum hysteresis errors can be calculated as follows:

(5) uh=(y)upscale−(y)downscale,

(6) $$\%uh_{max}=\frac{uh_{max}}{r_{O}}\cdot 100=\frac{uh_{max}}{y_{max}-y_{min}}\cdot 100,$$

where *uh* is the hysteresis error, (*y*)*_upscale_* is the upscale sequence test result, (*y*)*_downscale_* is the downscale sequence test result, *uh_max_* is the maximum hysteresis error, and *r_o_* is the full-scale output range. i.e., $r_{O}$ = $y_{max}-y_{min}$. The upscale sequence test result is the output value when acceleration increases from 0 to 3.6 g, and the downscale sequence test result is the output value when acceleration decreases from 3.6 g to 0. The hysteresis error is difference between (*y*)*_upscale_* and (*y*)*_downscale_*. The maximum hysteresis 15.04% was found at the acceleration of 0.9 g. Hysteresis error [63,64] seems to be caused by elastic after-effect. [65]

**Figure 11:**
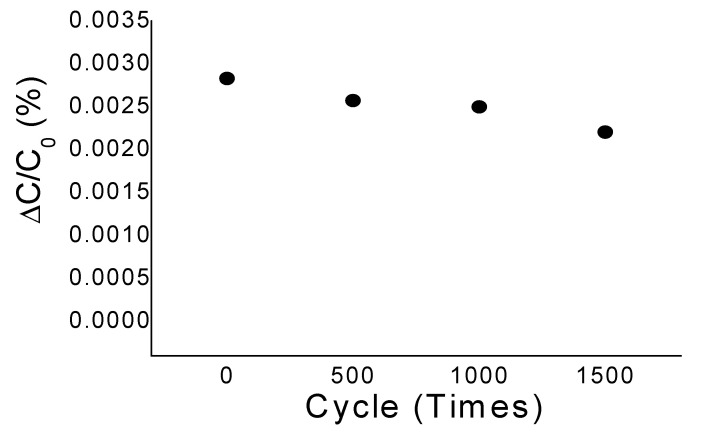
Capacitance change of the cantilever type acceleration sensor samples under the repeated occurrences of acceleration.

Finally, repeatability of the electrical performance of the sensor samples was examined (Figure 11). The samples were put under an acceleration of 3.6 g that was set to occur repeatedly with four different cycles (0, 500, 1000, and 1500). Average capacitance changes for all the cases at 3.6 g are compared. The experimental result shows that the average capacitance changes for the cycles of 0, 500, 1000, and 1500 are 0.028%, 0.026%, 0.025%, and 0.023%, respectively. This implies that the amount of change increases as the number of cycles does. In fact, the amount of change for 500, 1000, and 1500 cycles compared to 0 cycle is 7.14%, 10.71%, and 17.86%, respectively. The repeatability characteristic of the acceleration sensor thus needs to be improved, which can be obtained by employing materials with stronger restoration and elastic force than epoxy resin for the structural layer.

Compared to conventional MEMS air gap-based cantilever type capacitive acceleration sensors [66,67,68] and printed micro-sized acceleration sensors [69], the roll-to-roll slot-die coated sensor samples have lower electrical sensitivity and higher hysteresis error. This result is due to the relatively large size of the sensor samples. Since the electrical performance of our acceleration sensors is affected greatly by the air gap thickness, reducing the size of sensor samples is desired highly. In fact, the sensor samples made in this study are about 75% of our previous bridge type air gap-based touch sensors [55].

One of the most important obstacles to size reduction is stiction. The current roll-to-roll printing technology enables the printing of patterns with nanometer-sized width and thickness. Therefore, if the stiction problem is solved, printed sensor samples are expected to be made as small as conventional MEMS sensors. The authors suppose that various anti-stiction methods used in MEMS technology can be helpful for solving the stiction problem. For example, applying materials with higher resilience and restoration force to the structural layer can be a solution to the problem.

## 4. Conclusions

Air gap-based cantilever type capacitive acceleration sensors were fabricated on a flexible PET substrate using the roll-to-roll slot-die coating and the spin coating methods. Water-soluble PVA was used as a material for the sacrificial layer. The structures of the air gap and cantilever were fabricated by removing the sacrificial layer in water. The width and the thickness of air gap were 7 mm and 108.7 μm, respectively. The average sensitivity of the sensor samples for the range of acceleration from 0 to 3.6 g was 0.00856%/g. This study presents the possibility of fabricating air gap-based cantilever type acceleration sensors using the productive roll-to-roll process. The authors suppose the current acceleration sensor samples can be applied in the areas of mechanical, robotic, and wearable devices as motion sensors. After further optimization of design, materials, and processes, the application areas can be extended to those of conventional MEMS sensors. For the future work, the authors plan to reduce the size of the sensor samples to micro-scale by optimizing processes and materials in order to improve electrical characteristics. The authors also plan to fabricate acceleration sensor arrays that are suitable for sensing three dimensional acceleration.

## Figures and Tables

**Table 1:** Materials and fabrication processes for the cantilever type acceleration sensors.

Layer	Material	Process	Curing Condition
Bottom electrode	Ag	Roll-to-roll slot-die coating	Infrared oven 100 °C, 10 min
Dielectric	PMMA	Roll-to-roll slot-die coating	Infrared oven 70 °C, 10 min
Sacrificial	PVA	Roll-to-roll slot-die coating	Infrared oven 70 °C, 10 min
Spacer	BaSO4	Spin coating	Hot plate 150 °C, 30 min
Top electrode	Stretchable Ag	Spin coating	Hot plate 150 °C, 30 min
Structural	Epoxy resin	Spin coating	Hot plate 150 °C, 10 min
